# Differences in clerkship development between public and private Brazilian medical schools: an overview

**DOI:** 10.1186/s12909-020-02193-3

**Published:** 2020-09-21

**Authors:** Mauricio Braz Zanolli, Derly Silva Streit, Dione Tavares Maciel, Evelin Massae Ogata Muraguchi, Milton Arruda Martins, Iolanda Fátima Lopes Calvo Tibério

**Affiliations:** 1 Department of Internal Medicine, Marilia Medical School, Rua Comandante Romão Gomes, 33, Marilia, SP CEP: 17515-280 Brazil; 2 Petropolis Medical School, Petropolis, Rio de Janeiro State Brazil; 3 Pernambuco State University Medical School, Recife, Pernambuco State Brazil; 4 Londrina State University Medical School, Londrina, Parana State Brazil; 5 Medicine School of São Paulo State University, São Paulo, São Paulo State Brazil

**Keywords:** Internship and residency, Schools, medical, Education, medical, Educational measurement, Institutional evaluation, Infrastructure, Curriculum, Public medical schools, Private medical schools, Brazilian medical schools

## Abstract

**Background:**

Around the world, it is very expensive to become a physician. Although public medical schools are less expensive than private medical schools, tuition fees are charged at public medical schools in the majority of countries. In Brazil, public medical schools, with the exception of municipal schools, are free. There has been little investigation of any differences in conditions offered by paid or free medical schools or what occurs in public and private clerkships in Brazil. We investigated the clerkship conditions offered to the students in both public and private Brazilian medical schools by gathering the opinions of clerkship coordinators and others responsible for clerkships.

**Methods:**

A cross-sectional, descriptive, analytical study using an electronic questionnaire was answered by clerkship coordinators to compare the clerkships of 30 public and 38 private Brazilian medical schools from all regions of the country. The questionnaires covered various aspects of the clinical environments, student supervision, faculty development, student assessments, rotation evaluations and extracurricular activities developed by students.

**Results:**

We observed significant differences between public and private medical schools in several aspects investigated. Based on the opinions of the clerkship coordinators, with the exception of access to university hospitals, which was predominantly offered by public medical schools, private medical schools offer better clerkship conditions. The main differences were related to the number of positions, infrastructure, clinical learning environments, faculty development, student assessments, rotation evaluations and students’ extracurricular activities.

**Conclusion:**

This is the first study comparing Brazilian medical clerkships in private and public medical schools and provides a general vision of these programmes. It is necessary to further investigate clerkship development in the Brazilian medical school system and to study the differences between private and public medical schools globally.

## Background

“Public vs. private medical school: Which should you choose?” This was posted on the blog Wall Street Physician on April 9, 2018 (https://www.wallstreetphysician.com). The discussion was centred on American students’ apprehension in selecting a medical school as they considered medical school rankings, private or public schools, tuition, facilities with good residency programmes, how much money they would earn in their career, and how much student loan debt they would incur. The conclusion was that this decision must be very well evaluated. In August 2018, the New York University School of Medicine (NYUSM) initiated a programme offering full-tuition scholarships to all current and future students in its MD degree programme, regardless of need or merit, a bold effort to simultaneously address the rising costs of medical education and attract the best and brightest students to careers in medicine. NYUSM is the only medical school ranked in the top 10 in the United States of America (USA) to do so. Other similar programmes, available for selected students, are offered by others American and European Medical Schools [1–3]. Nonetheless, tuition-free medical education is unusual in the majority of countries around the word [4, 5]. In Brazil, however, with the exception of the municipal medical schools, all public schools are completely free of tuition fees. However, gaining access to these free medical schools is extremely difficult, as entry is granted based upon only one exam, “The Vestibular”. Hence, entry into public and highly ranked private schools is extremely challenging, and often, more than 200 candidates vie for each opening. In Brazil, the free public medical schools are funded by the federal and state government, the paid public municipal schools are frequently administered by a nonprofit municipal foundation, and the private medical schools are administered by nonprofit (primarily religious) or for-profit institutions. The average monthly tuition fees, in 2018, are approximately US$ 2000 (R$ 7000), ranging from R$ 3641.24 to R$ 12,738.06 (US$ 1000 to US$ 3500) [6]. Students who are not able to self-fund their education can receive financing, mainly offered by the Brazilian government. This financing can be repaid over a period of up to 18 years after graduation and at low interest rates [7].

In the past two decades, in an attempt to address the drastic shortage of physicians in remote, poor and dangerous areas, the Brazilian government authorised a mass expansion in the number of medical schools, mainly private schools, which today represent 63% of Brazilian medical schools. This expansion was stimulated by the “More Physicians Program” (Programa Mais Médicos) from the Brazilian Health and Education Ministries and resulted in triple the number of medical schools in Brazil [8]. Today, there are 334 medical schools in Brazil, offering a total of 34,748 positions each year. While the number of medical schools in the USA increased by 18% (125–147) and the number of students increased by 29% (16,844–21,806) in the past 20 years, in Brazil, the increase in this time period was more than 200% (100–334), with an increase from approximately 10,000 to 34,748 students [1, 9, 10].

Differences between private and public schools are rarely investigated, not only in Brazil, but also around the world. Washko et al. found that in the USA, more physicians electing to work in primary care and in rural areas graduated from public medical schools than from private medical schools [11]. In India, Diwan et al. found that there was little difference in the background characteristics, motives for entering the field of medicine or career aspirations between medical students from public and private institutions [12]. In a study conducted in 2004 on 88 American medical schools, Kamim et al. found no differences in the number of infrastructure features or services offered based on the region of the country, the size of the graduating class or whether a school was public or private. We did not find studies on differences between private and public medical schools in other countries around the world. It is important to note that in the majority of countries, public medical schools are not entirely free. Thus, studies on this topic must define the standards used to compare the medical schools. These standards must be based on official guidelines from the government or from medical education associations or consortiums such as the Brazilian Education Ministry, the Brazilian Medical Education Association, the World Federation for Medical Education, the Association for Medical Education in Europe, the American Association of Medical Colleges, the Scottish Deans’ Medical Education Group (The Scottish Doctor) or the German Association for Medical Education (The Frankfurt Model to ensure quality in teaching and learning) [13, 14]. Another important consideration about the standards is that they should agree with the national and/or international accreditation systems. In Brazil, we have had the Accreditation System of Medical Schools (SAEME, Sistema de Acreditação de Escolas Médicas) since 2016, which is organised and coordinated by the Brazilian Medical Education Association (ABEM, Associação Brasileira de Educação Médica) and the National Council of Medicine (CFM, Conselho Federal de Medicina) and has been recognised by the World Federation for Medical Education since 2019.

Although Brazilian reports about clerkships in the rotation system, from the São Paulo State University Medical School and Rio de Janeiro National Medical School, were published as early as 1956, the clerkship as the final phase of the medical course was mandated only in 1969 by the Federal Education Board as an obligatory special rotation learning phase for all Brazilian medical courses [14, 15]. Currently, Brazilian clerkships are regulated by the National Curricular Guidelines for Medical Courses that mandate rotations in the areas of internal medicine, surgery, paediatrics, gynaecology/obstetrics, mental health and public health. The stipulated duration is 2 years, and 30% total must be completed in primary care (mainly) and in urgent/emergency care [16].

The learning environment is one of the factors that contribute to knowledge acquisition and the formation of a professional identity, particularly during the clerkship [17]. In addition, independent of what institutional organizers say to students at orientation, students’ environments have a greater impact on their professional development. National and international medical education associations must promote meetings and conferences focused on clerkship development were held to help institutional organizers create a clinical environment where students may fully develop their professional identity [18].

The training offered in medical school varies greatly among the free and paid medical schools throughout the world, particularly in Brazil, and it is necessary to investigate these differences in order to help students choose which medical school is best for them and to help medical school administrators improve their courses. Few studies have been conducted on clerkships in Brazilian medical schools. The first study, which was conducted in 1981 and involved local visits to all 75 medical schools in Brazil, detected many problems with clerkship development [19]. Another study, conducted from 2012 to 2014, by the Brazilian Medical Education Association, used an electronic questionnaire to generate an overview survey about the main aspects of Brazilian clerkship development, considering the Brazilian Education Ministry Guidelines for medical courses and the bases of the Experience Based Learning about “How and what do medical students learn in clerkships” [10, 20–23].

The objective of this paper will be to analyse the results of this questionnaire, identifying possible differences between private and public (free of charge) medical schools in the development of the clerkships.

## Methods

This is a cross-sectional, descriptive and analytical study developed from a questionnaire answered by clerkship coordinators from 68 of the 160 Brazilian medical schools with current clerkships at the end of 2012. Although only 42.5% of the schools invited submitted responses, medical schools from all regions of the country are represented, and the proportion of private and public schools is similar to that of the entire country.

The survey was developed by Brazilian Medical Education clerkship experts based on the Brazilian Curricular Guidelines for Medical Schools [21] and using the model of the experience-based learning (ExBL) in the clerkship, as suggested by Dornan and colleagues. The ExBL is a middle-range theory that is consonant with the higher-range communities of practice theory and is a theory of how medical students develop the identity of doctors during their progression from medical school entrant to qualified doctor [10, 22–25]. The SAEME standards were not used because they went into effect in 2016, after the research was completed.

The survey addressed general aspects of the medical school and specific characteristics of the clerkship development: management; infrastructure and diversity of teaching and learning scenarios; number of patients assisted by students; presence of multiple professional sharing sessions with other students and residents; presence of electives; student support; total, weekly and on-duty workload; supervision; faculty development; students’ assessment; clerkship evaluation; clerkship handbook; training in Advanced Trauma Life Support (ATLS), Advanced Cardiovascular Life Support (ACLS) and Paediatric Advanced Life Support (PALS); hidden curriculum; students’ extracurricular activities; and residency exam courses. These themes were distributed into 47 items. The draft survey was revised following two pilot tests: one on-site test performed at the Brazilian Medical Education Congress that was answered by administrators, professors and students, and another conducted online that was answered by medical education experts from different private and public medical schools. The suggestions were analysed and the appropriate corrections were made before the survey was administered.

Statistical analysis was performed using chi-square tests, Fisher’s exact tests or Fisher-Freeman-Halton exact tests. The “Epi Table” module from the Epi Info 6.04d software (Centers for Disease Control and Prevention, USA, 2001) was used for the calculation of chi-square and Fisher’s exact probabilities. For the Fisher-Freeman-Halton exact test, the probabilities were calculated using the online tool “VassarStats: Website for Statistical Computation” at http://vassarstats.net/. For 2 × 3 tables, the link is http://vassarstats.net/fisher2x3.html, and for 2 × 4 tables, the link is http://vassarstats.net/fisher2x4.html77-82. In all conclusions obtained by inferential analyses, the alpha significance level was set at 5% (α = 0.05) [26–31].

The project was approved by the Ethics in Research Committee of the São Paulo State University Medical School and registered on the Brazilian Platform (Plataforma Brasil) with the Presentation Certificate for Ethics Appreciation (Certificado de Apresentação para Apreciação Ética - CAAE) number 58929915.8.0000.0065 on 29 August 2016.

## Results

Of the 160 Brazilian medical schools invited to participate, 68 responded (return rate of 42.5%). The sample was representative of Brazilian medical schools, including the proportion of public unpaid (30) and private paid (38) schools, as well as regional distribution of the schools.

The number of students enrolled each year (positions) ranged from 30 to more than 120, with a median of 100 positions annually. There was a significant difference (*p* = 0.023) between public and private schools, with between 96 to 120 positions in private schools and more than 120 positions in public schools. Nowadays, according to the site https://www.escolasmedicas.com.br, these numbers have changed since the survey was conducted, and most of the schools, mainly the private schools have increased their number of positions.

With regard to clerkship management, only half of the medical schools had a clerkship committee, and only 13% involved students in this task. In the other schools, clerkship management was undertaken by the course coordinator or the medical school director. There was no significant difference between private and public schools.

There were no significant differences between private and public medical schools regarding clinical experiences offered at secondary hospitals, urgent/emergency care units, primary care units, ambulatory centres, learning and simulation laboratories, and psychosocial support centres. However, 21% of schools did not have tertiary/quaternary (university/academic) hospitals, and there was a significant difference between private and public medical schools, as shown in Table 1. As we can see this is more frequent in private medical schools that have more partners that own 3°/4° hospitals.

**Table 1:** Learning Scenarios: Tertiary/Quaternary Hospital

3°/4° Hospital	Public	Private	Total	***p***-value
**Did not have one hospital**	3	11	14	**< 0.001**
**Only Own**	21	7	28	
**Only Partners**	0	16	16	
**Both**	6	4	10	
**Total**	30	38	68	

p = bilateral probability of the Fisher-Freeman-Halton exact test

Patient volume for students was considered to be adequate by 87% of the private schools and 67% of the public schools; thus, there was a significant difference (*p* = 0.02) between public and private schools. No private schools responded that patient volume was inadequate, while 17% of public schools reported inadequate patient volume.

Considering the infrastructure, there were significant differences between private and public medical schools, as shown in Table 2. It is important to emphasise that, in the opinion of private medical school clerkships, the infrastructure was never deemed inadequate.

**Table 2:** Infrastructure Adequacy: Physical, Technological and Human Resources

Infrastructure	***Adequacy***	Public	Private	Total	***p***-value
**Physical Space**	*Complete*	6	19	25	**0.008**
	*Partial*	21	19	40	
	*Inadequate*	3	0	3	
	*Total*	30	38	68	
**Equipment**	*Complete*	7	20	27	**0.009**
	*Partial*	20	18	38	
	*Inadequate*	3	0	3	
	*Total*	30	38	68	
**Human Resources**	*Complete*	6	23	29	**0.001**
	*Partial*	23	15	38	
	*Inadequate*	1	0	1	
	*Total*	30	38	68	

p = bilateral probability of the Fisher-Freeman-Halton exact test

The clerkship scenario shared with other students from the same course or other secondary- or tertiary-level health courses and residents from the same or other public or private institutions was present in most medical schools. There was a significant difference in the prevalence of sharing with residents from other private institutions (15 schools total), with 89% of private schools considering this scenario to be helpful and beneficial versus 33% of public schools viewing this scenario positively. There was also a significant difference in opinion on sharing with university students from other health care providers’ carriers and public institutions, which occurs in 17 medical schools: 88% of the public schools believe that this is helpful versus 11% of the private schools.

In the majority of schools, the clerkship period was 24 months, and they permitted elective/optional rotations inside and outside of the institutions. Few schools offered financial or other student support. No significant differences in these aspects were found between private and public schools.

Regarding theoretical activities, most schools (65%) offer various activities, such as classes, courses and clinical case discussions, as well as journal clubs, pathological/anatomy sessions, clinical discussions in small groups, team-based learning, laboratory activities, ethical discussions, etc. In 91% of private versus 44% of public schools, there was sufficient time for these other activities, and this difference was significant (*p* = 0.002).

No clerkship handbook was available in 30% of public and 8% of private schools, and this difference was significant (*p* = 0.023).

Rotations were undertaken in the main areas, as recommended by the government. The weekly workload was approximately 40 h, with 12 h on duty, and without differences between private and public schools. Regarding the duration of the rotations, there was a significant difference (*p* = 0.001) in short rotations (3 weeks or less), which were present in 37% of the public schools and only 5% of the private schools.

Student supervision was overseen mainly by academic teachers and attending physicians, multi-professional team health workers and even by residents. These professionals also undertook attending functions and other student supervision activities. There were no significant differences between private and public schools in the supervision items.

Faculty development programmes were not present in several schools. Programmes for academic teachers were present in only 40% of public and 68% of private schools, showing a significant difference (*p* = 0.024). Faculty development was available for attending physicians (33%) and other health profession workers (10%) and was less common for residents (1.5%) and clerkship students (4%), without significant differences between public and private institutions.

Concerning the students’ assessments, we detected significant differences. We observed differences in the prevalence of formative and summative assessments with feedback between the private and public medical schools (69 and 41%, respectively; *p* = 0.023); in the prevalence of both summative and formative students’ clerkship assessment instruments in the private and public schools (97 and 59%, respectively; *p* <  0.001); and in the prevalence of student assessment feedback in private and public medical schools (97 and 76%, respectively; *p* = 0.017; Tables 3 and 4).

**Table 3:** Clerkship student assessment instruments

Instrument	Public	Private	Total	***p***-value
**Only Summative**	6	1	7	**< 0.001**
**Only Formative**	6	0	6	
**Both**	17	35	52	
**Total**	29	36	65	

p = bilateral probability of Fisher-Freeman-Halton exact test

**Table 4:** Feedback for Student Assessments

Feedback	Public	Private	Total	***p***-value
**Yes**	22	35	57	**0.017**
**No**	7	1	12	
**Total**	29	36	65	

p = bilateral probability of Fisher’s exact test

The student assessments were conducted using different kinds of instruments, including cognitive tests, objective structured clinical examination (OSCE), a portfolio either from each rotation or at the end of the year and qualitative direct observation by a supervisor. The only method with a significant difference (*p* = 0.007) was the supervisor qualitative direct observation, which was used in 90% of the public schools versus 61% of the private schools. Student educational recovery was not offered by 63% of public and 29% of private schools, and this difference was significant (*p* = 0.022).

Rotation evaluations were present in all of the private schools and in only 43% of the public schools; this difference was significant (*p* <  0.001; see Table 5). The rotation evaluations were conducted at different intervals with different types of instruments, but feedback was performed in only 30%.

**Table 5:** Rotation Evaluations

Evaluations	Public	Private	Total	***p***-value
**Yes**	21	36	57	**< 0.001**
**No**	9	0	9	
**Total**	30	36	66	

p = bilateral probability of the Fisher’s exact test

The students participated in different extracurricular activities, including the Students’ Scientific Association, residency preparatory courses, voluntary duties, scholar medical exams and activities with the instructors outside of medical school. Activities with instructors outside of medical school were performed more in private (47%) than in public (21%) medical schools, and the difference was significant (*p* <  0.05).

## Discussion

The characteristics of medical clerkship in Brazil have not been adequately explored, and the differences in clerkships between private and public schools are understudied not only in Brazil, but also around the world. As we presented in the Introduction, it is very important for students to know the characteristics of the medical course in public and private medical schools, including their clerkships, and also to know how much money will be necessary for the course. This is important for the students’ decisions regarding their social and economic situations, as well as perspectives for their future careers, medical specialisations and where they intend to work after graduation [32].

In our study, we found significant differences between public and private Brazilian medical schools in several aspects of clerkship development. The main aspects in which differences were found are related to the number of positions, infrastructure, clinical learning environments, faculty development, student assessments, rotation evaluations and students’ extracurricular activities.

In the literature, there are few publications comparing private and public schools that consider the conditions offered to students in the graduation process. In our research, we detected differences in the opinions of the clerkship coordinators in 29% of the aspects investigated. There was a tendency for the opinion of the clerkship coordinators in the public schools to be more critical, relative to the items researched, as overall, they are of the opinion that the conditions offered to the students in the clerkships are more unsatisfactory than those in the private schools.

In our study the number of positions offered each year, for each school, in Brazilian public medical schools tends to be higher than those in private schools, but as the number of private schools are higher, the total the number of positions, also is higher. In the USA, according to Barzansky and Etzel, in the academic year 2017–2018, there were 21,806 positions in 147 medical schools, with 63% of those in public schools, which is the opposite of Brazil (however, we must consider that public medical school is totally free in Brazil, and in the USA, it usually is not) [33]. Currently, the situation has changed, as we acknowledged in the introduction. With the increasing number of medical schools in Brazil, mainly private schools, the yearly number of training slots offered has also changed, and this change has been more substantial in private medical schools.

Relative to the infrastructure for the clerkship development, the private schools offer fewer high-complexity hospitals to the students, although those responding to the questionnaire all reported that their infrastructure and the number of patients with whom the students have contact is satisfactory. In United States medical schools, Kamim et al. did not find differences in the infrastructure and services offered by private and public medical schools [13]. In the majority of medical schools, the practical learning scenarios are shared with other students and residents, and few differences are seen between private and public schools regarding whether they view this as a help or a hindrance to the students’ learning. In fact, in Brazil, as in the USA, primarily due to the large increase in medical schools and in positions offered, clerkship scenarios are being shortened, mainly in the healthcare system, resulting in less time in the hospitals, urgent/emergency units, ambulatory centres and primary care centres. In Brazil, as in the USA, the competition between private and public schools is increasing, and frequently, as the private schools possess more resources and facilities to pay as many supervisors/preceptors as are needed, the public schools are sometimes negatively impacted. It is necessary to expand the undergraduate clinical teaching capacity to offer good learning conditions for medical students, particularly in their clerkship [34–38].

In addition to the hands-on teaching directly with patients, medical schools offer different teaching and learning instruments over and above traditional courses, from classes and clinical case discussions to other activities such as journal clubs, anatomy/pathology sessions, team-based learning, laboratory activities and ethical discussions. The private schools reported that they have sufficient time for the majority of other activities, but less than half of the public schools reported the same; however, the significance of this is difficult to interpret, as we did not investigate the time dedicated to these activities. Many different learning strategies with very good results are described in the literature, including virtual environments, team-based learning and simulation training (mainly life support) [39–41]. The use of problem-based learning in the clerkship is that is difficult to apply, and some problems have been reported for this method [42–44].

Clerkship handbooks could support students by providing more information about clerkship development, timetables and assessment, among other activities, as was demonstrated by Atherley and Taylor Jr. at the University of the West Indies Faculty of Medical Sciences in Barbados [45]. Unfortunately, in Brazil, 30% of public medical schools do not offer this to their students, creating confusion amongst students and a lack of understanding as to exactly what they are required to do and learn in each rotation during their clerkship.

We found that rotations are the same in almost all schools, and the only significant difference we identified is the presence of short rotations (3 weeks or less) in public schools. The longitudinal integrated clerkship model is proposed as a very good option when compared with block rotation models, as this model permits more integration of instructors, learners and patient relations [46–48]. Comparing two- and four-week rotations, Elnicki and Cooper found that shorter rotations did not negatively impact the students’ experiences, but that evaluations were difficult; on the other hand, Lucas et al. concluded that shorter rotations are worse according to trainees’ evaluations [49, 50].

Brazilian medical schools’ faculty development programmes are unsatisfactory, particularly in public schools, although the importance of these programmes is very clear in the medical education literature and it is very nicely demonstrated in the book by Harden and Lilley, *The Eight Roles of the Medical Teacher* [51].

A few different kinds of instruments were used for student assessments in Brazilian medical schools. In the opinion of private school clerkship coordinators, these institutions more frequently use both summative and formative instruments and feedback. Although Terry et al. suggested that OSCE may be the most appropriate summative assessment for educators to identify students who may be at risk of poor performance in a clinical workplace environment [52]; only 30% of Brazilian medical schools used OSCE. These results show that we need to improve student assessment, mainly in public schools, using more feedback and new skills assessment instruments, as presented in the papers of Walls et al. and Norcini et al. in 2018; the consensus framework is suggested for good assessment [53, 54].

The clerkship development evaluation is also unsatisfactory, particularly in public schools, as 30% of them do not have any kind of evaluation. Rotation evaluations are very important, but we first need to define rotations as a learning environment. Gruppen et al. suggested a conceptual framework of the learning environment that identifies five overlapping and interactive core components that form two dimensions: the psychosocial dimension and the material dimension. The authors offered several practical suggestions for health profession educators, investigators and editors, including the evaluation of the environments in order to improve student learning [55]. Stalmeijer et al., in the Netherlands, developed a cognitive apprenticeship model that appears to offer a useful framework for the development of an evaluation instrument aimed at providing feedback to individual clinical teachers on the quality of their student supervision [56]. There are other instruments that can be used in the evaluation of the teaching and learning environment and climate. These mainly evaluate the clinical aspects. Examples include the Dundee Ready Education Environment Measure, with 50 items, and more recently, the Manchester Clinical Placement Index, which has only 8 items and is more specific to the clinical environments that are used in the clerkships. These instruments can be used to compare clerkship development in different medical schools [57–59].

In Brazil, Pontes et al. found that after a new curriculum was introduced at Paraiba Federal University, student satisfaction with their clerkship was inadequate, including several aspects of student learning, learning environments, resident relationships and teacher roles [60]. Two other papers analysed the same aspects in the Primary Healthcare Clerkship in Santa Catarina and Alagoas Federal Universities and reported that the students’ opinions about the experience were favourable and that adjustments may be made to improve and strengthen teaching and service integration [61, 62].

Regarding the hidden/parallel curriculum developed by Brazilian clerkship students, there are a few differences between the private and public schools. The predominant difference is that the students in private schools participate in more activities with their instructors outside of their university jobs, which is very common among Brazilian medical school instructors. These activities are conducted to complement the students’ learning, mainly in clinical skills, and perhaps take place more in private medical schools where part-time instructors are more frequent. The results of a study conducted at Minas Gerais Federal University Medical Faculty (public) by Tavares et al. showed that the “parallel curriculum” was a reality for most of the students: 82.5% participated in extracurricular activities, dedicating an average of 8.2 h per week to these activities. The primary motivation for participating in these activities was achieving more practical experience and improving upon the curriculum [63]. As residency admission in Brazil is determined mainly by means of knowledge on multiple choice question (MCQ) exams with a very high level of failure, most students attend residency preparatory courses in the final two and sometimes 3 years of medical school. The effect of these courses, offered to provide training to students in answering MCQ exams, not to increase their learning, can disturb the students’ clerkship learning and, in addition, result in a high financial cost to the students. The economic implications of and results from residency admissions tests, as well as their effects on clerkship learning, are very controversial and have been discussed in papers in the Brazilian Medical Education Journal [64–67] and in the New England Journal of Medicine [68].

Regarding students’ test results upon graduation, although not investigated in our research, in the volunteer participation exam administered at the end of the course by the São Paulo State Medical Council, students from public medical schools achieved better results in the past 10 years [69]. We must note here that Brazil does not have official final certification exams [70].

The differences found may reflect differences in the conditions offered by public and private schools, mainly due to financial resources, which is currently a critical situation in Brazilian public medical schools. These resources can influence in the infrastructure offered by the public schools. Often, the salaries are lower in the public medical schools, which may influence the motivation of instructors and technical personnel.

This study has some limitations, with the primary limitations being that the questionnaires were answered by the clerkship coordinator/administrator, without involving the opinions of the students and teachers and this could result in a reporting bias for private and public medical schools opinions. Although only 42.5% of schools invited answered the questionnaire, the participating schools were representative of all Brazilian medical schools from all regions and had the same proportion of public and private schools. Another limitation was that the survey elaboration was based mainly on the Brazilian Guidelines; therefore, reproducibility in others countries is limited. Moreover, no formal validation method was done. Finally, we did not use accreditation standards of the SAEME because these standards did not go into effect when the study was conducted. We agree with the statement of Professor Roger Peter Strasser, from Northern Ontario School of Medicine that “medical school accreditation is the means by which the differences reported in this paper are addressed to ensure consistent high quality of medical education in all schools whether they are public or private”.

The study also has many strengths. In particular, we created a snapshot of the clerkship developed in the Brazilian medical schools prior to the new guidelines suggested by the Brazilian Educational Ministry in 2014, which can be used for future studies to analyse the impact of the changes made after the implementation of these new guidelines in Brazilian medical education. In addition, this is the first study comparing the development of Brazilian medical clerkship in private and public medical schools.

## Conclusions

There is a dearth of literature regarding the comparison between private and public medical schools, perhaps because, globally, the concept of private and public medical schools is diverse and mainly related to whether the students are required to pay tuition fees.

Our study provides a general view of the Brazilian clerkships and the differences in clerkships between private (paid) and public (free) schools. We found that, in the majority of aspects investigated, clerkship administrators were of the opinion that the conditions offered to students in private medical schools are superior. This likely reflects the better financial conditions in private medical schools. The main aspects in which differences were identified were related to the number of positions, infrastructure, clinical learning environments, faculty development, student assessments, rotation evaluations and students’ extracurricular activities.

The analysis of the differences, as well as the drawing of conclusions, must be done very carefully, as it is probable that the private medical schools, especially those with very high tuition fees, possess better financial conditions to offer a superior infrastructure (particularly with teaching and learning labs). In some cases, they do face challenges in access to hospitals, mainly university hospitals. On the other hand, public schools have more competition in admissions and may have more competitive students. It is necessary to conduct further research to better understand the differences between private and public schools in all the aspects involved in clerkship development in Brazil and worldwide, using the standards of the national and international medical education associations. It is also necessary to investigate the influence of different clerkships and medical schools on students’ performance, their medical specialty of choice and where the students choose to work after graduation (specifically, if they elect to work as primary care/family physicians). We hope that investigations of the differences between public and private medical schools, particularly with regard to clerkships, continue, both in Brazil and worldwide. We believe that our study may be one alert for medical schools managers to looking for some aspects that must be improve in the clerkship development in both private and public medical schools.

## Data Availability

The questionnaire and the data sets used and/or analysed during the current study are available from the corresponding author on reasonable request.

## Abbreviations

NYUSM: New York University School of Medicine
MD: Medical Doctor
USA: United States of America
UCLA: University of California at Los Angeles
SAEME: Sistema de Acreditação de Escolas Médicas (Medical School Acreditation System)
ABEM: Associação Brasileira de Educação Médica (Brazilian Medical Education Association)
CFM: Conselho Federal de Medicina (National Council of Medicine)
ExBL: Experience Based Learning
ATLS: Advanced Trauma Life Support
ACLS: Advanced Cardiovascular Life Support
PALS: Pedriatric Advanced Life Support
CDC: Center for Disease Control and Prevention
CAAE: Certificado de Apresentação para Apreciação Ética (Presentation Certificate for Ethical Appreciation)
OSCE: Objective Structured Clinical Examination
DREEM: Dundee Ready Education Environment Measure
MCPI: Manchester Clinical Placement Index
MCQ: Multiple Choice Questions
RBEM: Revista Brasileira de Educação Médica (Brazilian Medical Education Journal)
USMLE: United States Medical Licensing Exam
